# KL2 scholars’ perceptions of factors contributing to sustained translational science career success

**DOI:** 10.1017/cts.2021.886

**Published:** 2021-12-28

**Authors:** Susan S. Smyth, Barry S. Coller, Rebecca D. Jackson, Philip A. Kern, Scott McIntosh, Emma A. Meagher, Doris M. Rubio, Kathryn Sandberg, Joel Tsevat, Jason G. Umans, Jacqueline Attia, Heather L. Baker, Joan D. Nagel, Colleen A. McMullen, Erica Rosemond

**Affiliations:** 1 University of Arkansas for Medical Sciences, Little Rock, AR, USA; 2 Rockefeller University, New York, NY, USA; 3 The Ohio State University, Columbus, OH, USA; 4 University of Kentucky, Lexington, KY, USA; 5 University of Rochester, Rochester, NY, USA; 6 University of Pennsylvania, Philadelphia, PA, USA; 7 University of Pittsburgh, Pittsburgh, PA, USA; 8 University of Texas Health Science Center at San Antonio, San Antonio, TX, USA; 9 Georgetown University, Washington, DC, USA; 10 National Center for Advancing Translational Sciences, National Institutes of Health, Bethesda, MD, USA

**Keywords:** Career development, translational science, career success, career satisfaction, work impact

## Abstract

**Introduction:**

Identifying the most effective ways to support career development of early stage investigators in clinical and translational science should yield benefits for the biomedical research community. Institutions with Clinical and Translational Science Awards (CTSA) offer KL2 programs to facilitate career development; however, the sustained impact has not been widely assessed.

**Methods:**

A survey comprised of quantitative and qualitative questions was sent to 2144 individuals that had previously received support through CTSA KL2 mechanisms. The 547 responses were analyzed with identifying information redacted.

**Results:**

Respondents held MD (47%), PhD (36%), and MD/PhD (13%) degrees. After KL2 support was completed, physicians’ time was divided 50% to research and 30% to patient care, whereas PhD respondents devoted 70% time to research. Funded research effort averaged 60% for the cohort. Respondents were satisfied with their career progression. More than 95% thought their current job was meaningful. Two-thirds felt confident or very confident in their ability to sustain a career in clinical and translational research. Factors cited as contributing to career success included protected time, mentoring, and collaborations.

**Conclusion:**

This first large systematic survey of KL2 alumni provides valuable insight into the group’s perceptions of the program and outcome information. Former scholars are largely satisfied with their career choice and direction, national recognition of their expertise, and impact of their work. Importantly, they identified training activities that contributed to success. Our results and future analysis of the survey data should inform the framework for developing platforms to launch sustaining careers of translational scientists.

## Introduction

Developing, expanding, and sustaining the clinical and translational research workforce has been a major goal of the National Institute of Health (NIH) Clinical and Translational Science Award (CTSA). To date, all CTSA-supported institutions (hubs) have been required to have an institutional CTSA Mentored Career Development Award (KL2) to provide early career translational scientists from varied backgrounds with protected time and resources to develop high-impact translational science careers. The KL2 programs aspire to be exemplary models of career development programs.

Both the National Center for Advancing Translational Science (NCATS) and individual CTSA hubs have made significant investments in the KL2 program. More than 2,000 individuals have received support from KL2 programs since the program began in 2006. Additionally, over 60% of hubs commit additional institutional resources to expand the number of training spots beyond those provided by CTSA funds [1]. CTSA hubs provide research training opportunities that help KL2 Scholars develop the knowledge, skills and abilities needed to be successful in translational science, including career development experiences that promote the successful transition of KL2 Scholars to independent translational science careers. KL2 Scholars have at least 75% of their time protected for research and career development, unless they are in procedural disciplines, in which case the requirement is for at least 50% protected time, which must be requested from NIH. Additionally, KL2 Scholars receive up to $25,000 per year to cover costs related to tuition, research supplies, equipment, technical personnel, travel, workshops, externships, and statistical services.

The return on investment from the KL2 funding mechanism has been evaluated in two nationwide surveys of KL2 program directors [1,2]. The first survey in 2013 assessed the transition to independence of 914 KL2 Scholars, as measured by attainment of independent research funding [2]. At that time, 96% of the Scholars who had completed their KL2 training remained in research and 39% who had completed training 2 or more years earlier had received independent funding. A more recent survey in 2019 revealed that 78% of the KL2 program alumni were still participating in translational science, indicating a high retention rate in research following training [1].

While those studies assessed the impact of the KL2 program from the perspective of KL2 principal investigators (PIs)/program directors, only one study has focused on the organizational and personal factors reported by prior KL2 Scholars as contributing to career success. Using semi-structured interviews of 40 former K scholars, that study identified personal and organizational factors that were more common to former K Scholars who went on to obtain independent funding compared to Scholars who did not [3]. The goal of this study was to build on the previous findings by defining barriers and facilitators to sustaining careers in translational science as perceived by former KL2 Scholars and to obtain their assessment of the impact of various KL2 program components on their career progression and success. To this end, we conducted a survey of KL2 Scholar program alumni to gain information about their experiences both during and after their KL2 Scholar award period.

## Methods

The CTSA Program Steering Committee established a task force to identify obstacles to sustaining the careers of translational scientists. The task force designed a voluntary and anonymous survey that was pretested by content and survey experts, administered through the REDCap electronic data capture platform [4], and analyzed by the CTSA Center for Leading Innovation & Collaboration (CLIC) housed at the University of Rochester. The survey was reviewed by the University of Rochester Institutional Review Board and determined to be exempt as defined by Title 45 of the Code of Federal Regulations Part 46. The survey was sent via e-mail invitation (followed by two reminder emails) to 2144 KL2 alumni who had completed the KL2 program prior to 2019. Responses were collected between August and October 2019. The survey was comprised of both quantitative and qualitative questions. All responses were analyzed systematically and aggregated to preserve anonymity; all identifying information was redacted for reporting purposes.

Quantitative survey data were analyzed descriptively, with response choice proportions for categorical data and with means and standard deviations for continuous data per our established analysis and reporting strategies [5,6]. Logistic regression models were based on the variables: perception of success (“I feel my job is meaningful”), perception of job satisfaction (“How satisfied are you with the direction in which your career is progressing?”), and career stability (“From a financial perspective, how would you rate a career in Clinical and Translational Science Research?”). The predictors were outcomes and degree (MD, PhD, MD/PhD), years of stage/past training (3 or less, 4–6 and 7 or more), and if Male or Female. Some categorical responses of “Other” were re-organized for practical interpretation. Individuals who responded that they had applied for or received “Other (unspecified)” grants were re-categorized into established categories for which they met criteria and, for reporting purposes, their responses were re-classified to “Did not apply for this type of grant” (n < 60 participants). Additionally, responses of “Other Clinical” or “Other Research” terminal degrees were re-categorized into the corresponding MD degree category (n = 11). Instances in which responses were re-categorized are noted in the summary by an asterisk and notes on the transformation have been included in the corresponding supplementary data. All data are stored in both original and re-classified forms.

For the qualitative responses, 154 respondents generated 254 comments that were then coded by themes. Qualitative theme coding involved establishing a general framework for data analysis (open coding of text related to initial domains of interest), and an axial coding strategy based on the grounded theory approach [7], which led to specific categories. Two experienced coders independently assigned initial themes to each text response, followed by iterative team meetings to reach agreement on finalized themes, per procedures that we have used previously [5–9]. Inter-rater reliability was assessed using a simple proportion agreement rather than a more complex statistic due to the relatively large number of codes, the possibility for multiple codings within analyzable theme domains, and the exploratory nature of this study [10].

## Results

A total of 547 surveys were completed, representing a 25.5% response rate. Respondents had completed their KL2 program 1–12 years earlier, with 62.7% of respondents having finished within the previous 5 years. Nearly 80% of Scholar alumni had received two or three years of KL2 funding; 5.1% received four years and 3.5% received 5 years of support. Sixty percent of respondents were female, and 15.6% self-identified as an underrepresented minority in accordance with the NIH definition [11]. Very few respondents (1.1%) reported having a disability. A plurality of respondents (47.2%) had MD degrees, 36.1% had PhD degrees, and 12.8% had MD/PhD degrees; the remainder had other clinical or research degrees (e.g., PharmD, DVM, etc.). When first appointed to the KL2 program, 17.4% were Instructors, 65.3% were Assistant Professors, 8.6% were Research Assistant Professors, and 8.6% held other titles (e.g., postdoctoral fellow, fellow, staff scientist/research associate/research coordinator, and resident). During their KL2 funding period, 61.3% started or completed an advanced degree or certificate program; of those pursuing a degree, 80% reported enrollment in, or acquisition of, a Master’s degree, while others reported a certificate (10.4%), PhD (8.5%), or other degree (0.9%).

### Current Roles and Effort

At the time of survey completion, 95.2% of respondents had established careers in an academic setting and of these, 60.1% were tenured or on a tenure track, 24.7% were in non-tenure track research positions, 11.6% were in non-tenure track research and patient care positions, and 3.7% were in non-tenure track patient care positions. Of those not in academia: 0.9% worked in the pharmaceutical or biotechnology industries; 0.6% worked at the NIH, the Food and Drug Administration (FDA), or a not-for-profit organization; 0.6% were in private clinical practice; 0.2% were out of the workforce; and, 2.9% held other positions. About half of the participants (49.9%) also indicated that they held an administrative or leadership position.

Across all respondents, the distribution of current effort after the KL2 was on average: 50.6% for externally funded research, 23.5% for patient care, 17.5% for institutional/internally funded research, 10.9% for teaching, 10.9% for administration, and 7.7% for other activities. MD alumni reported spending approximately 60% of their time in research and 34% in patient care, whereas MD/PhD former Scholars spent approximately 70% of their time in research and PhD former Scholars spent approximately 80% of their time on research. Nearly half (47.4%) of all KL2 alumni reported serving as PI or multi-PI (MPI) on at least one research grant while 16.4% reported serving as a collaborator and 2.4% reported serving as a consultant on extramurally funded awards. At the time they completed the survey, both the MD and PhD cohorts reported having 47.3% of their effort supported as PI or MPI and similarly, the MD/PhD cohort reported 50.0% of their effort was supported as PI or MPI.

### Funding and Research

**Fig. 1.**
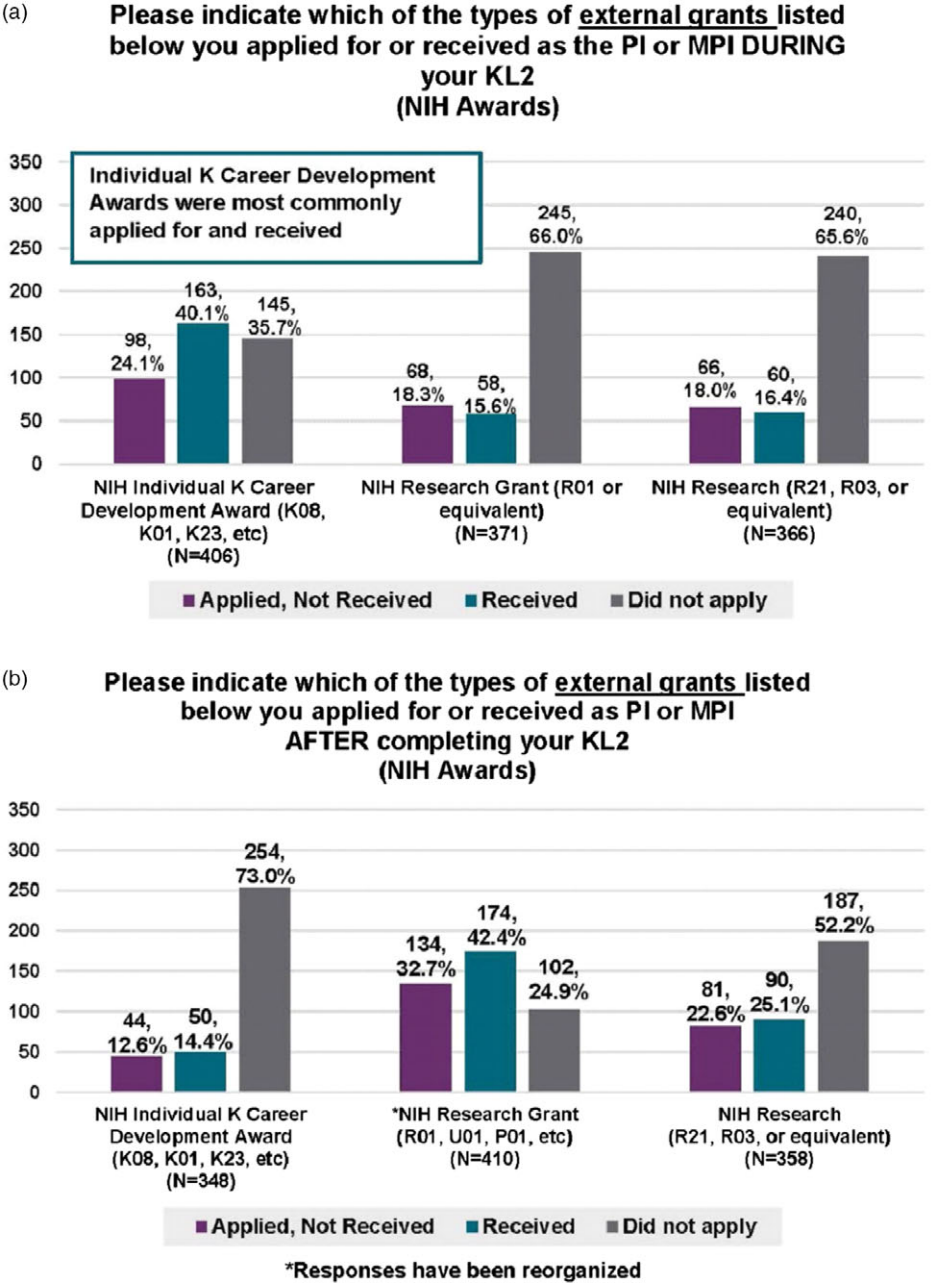
Grants applied for and awarded at 1–12 years after KL2 training. Based on data from 489 Scholars. A: N = 543 with a missing N = 4 (Yes= 454, No=89). B: N = 542, missing N = 5 (Yes= 443, No= 99). Results are presented as the percent of individuals that applied for and did not receive (purple), received (green), or did not apply for (gray) different types of grants by category. The number of respondents is indicated on top of the bars. Abbreviations: Clinical and Translational Science Award (CTSA) Mentored Career Development Award (KL2), National Institute of Health (NIH), Principal Investigator (PI), Multi-Principal Investigator (MPI), Research Grant (R), Career Development Awards (K).

During their appointment on the KL2 award, 83.6% of the Scholars applied for extramural grant funding. The type of NIH funding most commonly applied for and received was NIH individual career development (K) awards (64.5% applied, 40.1% received), followed by Research Project (R)21 (34.4% applied, 16.4% received) and R01 awards (33.9% applied, 15.6% received) (Fig. 1A). MD and MD/PhD Scholar alumni were more likely to apply for career development awards than were PhD Scholars (50.0% versus 28.4%; p = 0.01), whereas PhD Scholars were more likely to apply for R01s (25.0% for PhDs versus 6.9% for MDs and 15.7% for MD/PhDs; p = 0.01). Foundation grants were the most common non-NIH funding applied for and awarded (44.5% received). Other non-NIH funding obtained during the KL2 support period included industry-sponsored awards (23% received) and career development awards from the Department of Veterans Affairs (VA) or other national organizations (12% received). KL2 alumni also reported applying for local CTSA pilot awards (21.7%) and other institutional pilot funding awards (23.9%) while in the KL2 program.

After their KL2 appointment ended, approximately 82% of alumni applied for extramural grant funding as a PI or MPI. At this career stage, NIH research grants (R01 followed by R21 or equivalent) were those most commonly applied for and awarded, with 42.4% receiving an NIH R01 or similar research grant (Fig. 1B). A larger percentage of PhD alumni (50.6%) received R01 awards after their KL2 support than did MD (36.9%) or MD/PhD (39.2%) alumni (p = 0.01). Among the non-NIH grant support received, 46% of respondents reported funds from foundations/organizations (local or state), 8.3% from the VA and related organizations, and 15% from industry. Additionally, 45.4% of the KL2 alumni reported receiving intramural funds. Most respondents (83.2%) reported serving as a collaborator on an NIH grant after their KL2 ended. Of note, numerous Scholars reported receiving more than one type of award.

When asked to describe their science, respondents considered themselves to be a clinical researcher (55.5%) and/or a translational researcher (46.5%). Approximately half of the respondents (50.5%) thought that the broad goals of their current research are best described as “design and implementation of new health care delivery paradigms, best practices, treatment guidelines, or education programs to improve health outcomes.” Other broad research goals included: “understanding the pathophysiology of disease” (47.6%), “developing new drugs or devices or repurposing existing drugs” (28%), “developing new research methods” (18.9%), “improving understanding of basic biological processes” (18.7%), or “improving health or social policies” (15.2%); the remainder did not specify their research goals (8.1%) or were no longer conducting research (2.2%).

### Career Satisfaction and Work Impact

A total of 80.4% of respondents were satisfied with the direction in which their career was progressing; no difference was observed between women and men, by year since the KL2 ended (≤3, 4–6 years, and ≥7 years) or by degree type. Fully 95.0% of former Scholars believed that their current work was meaningful. Respondents with an MD degree were 2.4% more likely (P < 0.05) than other degree holders to indicate that their work was meaningful. Additionally, 76.1% of respondents “agreed” or “strongly agreed” that they were nationally recognized for their expertise in their scientific area, whereas 15.6% were “neutral” and 7.0% “disagreed” or “strongly disagreed” with that premise. Importantly, KL2 alumni identified categories across their translational spectrum where their scholarship had impact, particularly contributing to “health care delivery paradigms, best practices/treatment guidelines, educational programs” (42.0%); “understanding pathophysiology of disease” (40.9%); “developing new drugs, devices, including repurposing of existing drugs and devices” (17.6%); “improving understanding of basic biologic processes” (16.9%), “development of new research methods” (15.6%), “improving health policy or social policy” (13.0%), and other areas (5.9%). Only 3.9% of respondents felt that their work had not been impactful.

Sixty-six percent of respondents felt confident or very confident in their ability to sustain a career in clinical and translational science, although 81.3% of respondents reported that it is “challenging” or “very challenging” to hold multiple roles (i.e., educator, researcher, and/or clinician). When asked the extent to which they were satisfied with their work/life balance, 49.1% responded that they were “satisfied” or “very satisfied,” 28.5% were “neutral,” and 21.4% were “dissatisfied” or “very dissatisfied.” Sixty percent “agreed” or “strongly agreed” that they were paid fairly, 17.7% were “neutral,” and 22.0% “disagreed” or “strongly disagreed.” Similarly, half (50.5%) of the Scholars felt that a career in clinical and translational science was financially “very sound” or “reasonably sound,” with 21.4% being “neutral,” and 25.4% responding that it was not; no difference was observed between women and men, by year of training (≤3, 4–6, or ≥7 years) or by degree type.

### Impact of KL2 Opportunities on Career Success

**Fig. 2.**
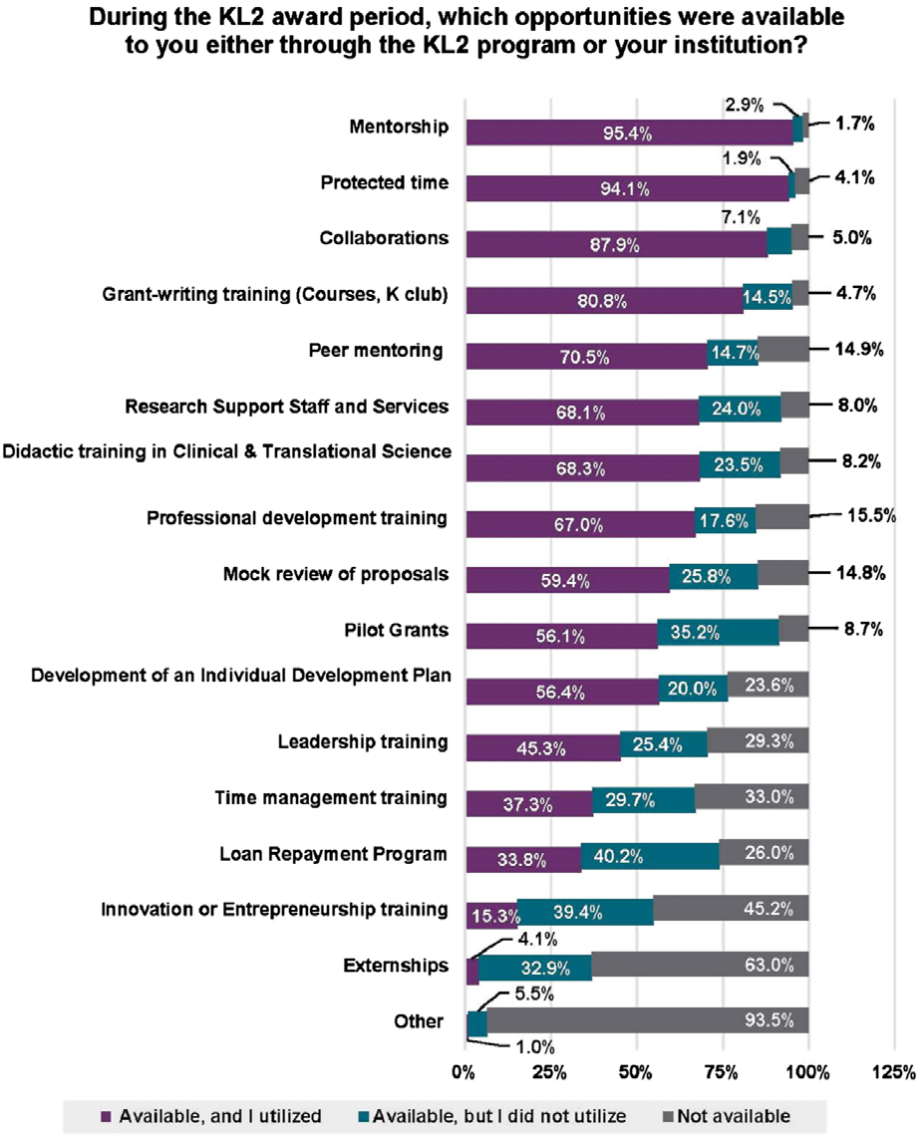
Opportunities available for career development. Opportunities were scored as being available and utilized (purple), available and not utilized (green) or not available (gray). Results are reported as the percent of total responses to each opportunity. Abbreviations: Clinical and Translational Science Award (CTSA) Mentored Career Development Award (KL2), Career Development Awards (K).

**Fig. 3.**
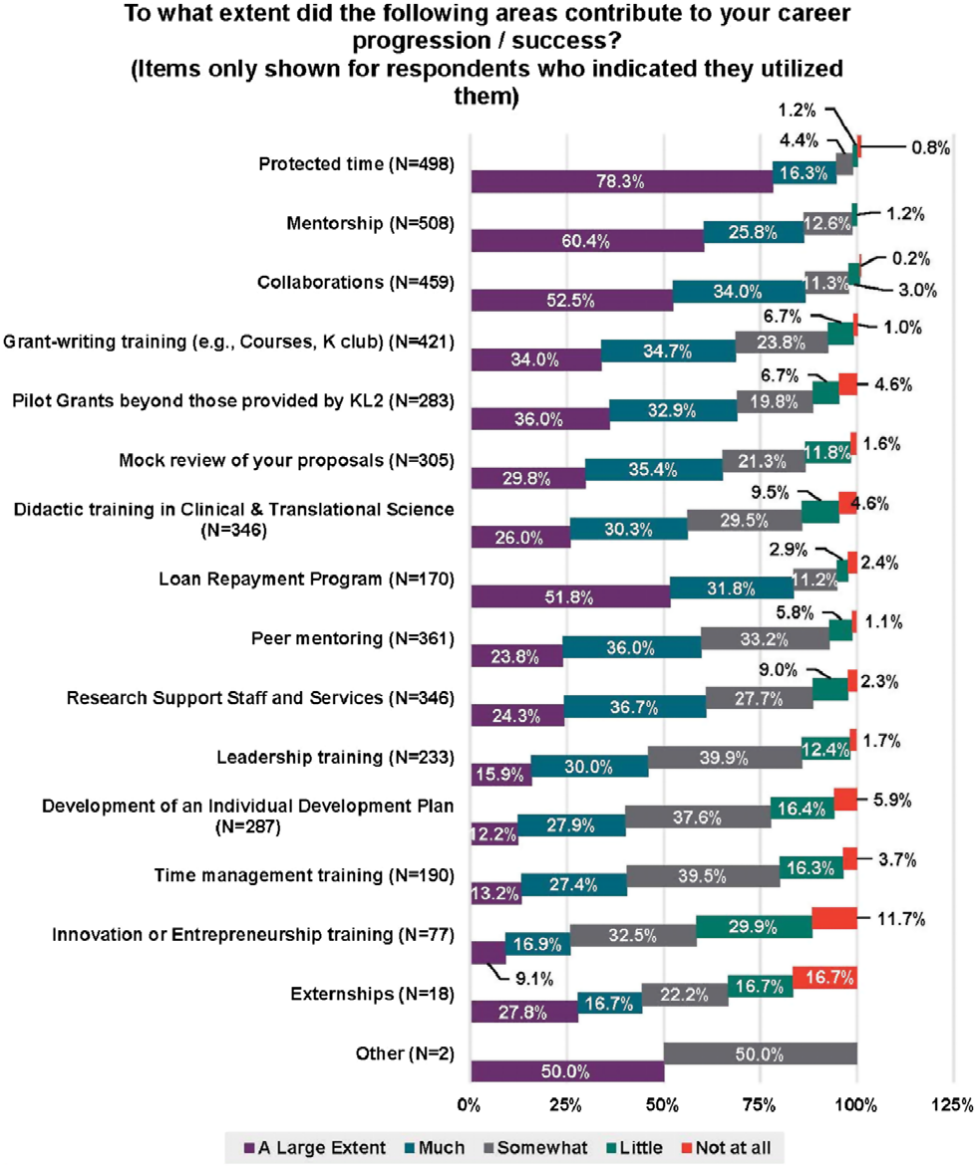
KL2 program features contributions to career success. The data are presented as the percent that felt the area contributed a large extent (purple), much (turquoise), somewhat (gray), little (green), or not at all (red). The number of responses for each area is indicated by N. Abbreviations: Clinical and Translational Science Award (CTSA) Mentored Career Development Award (KL2), Career Development Awards (K).

**Fig. 4.**
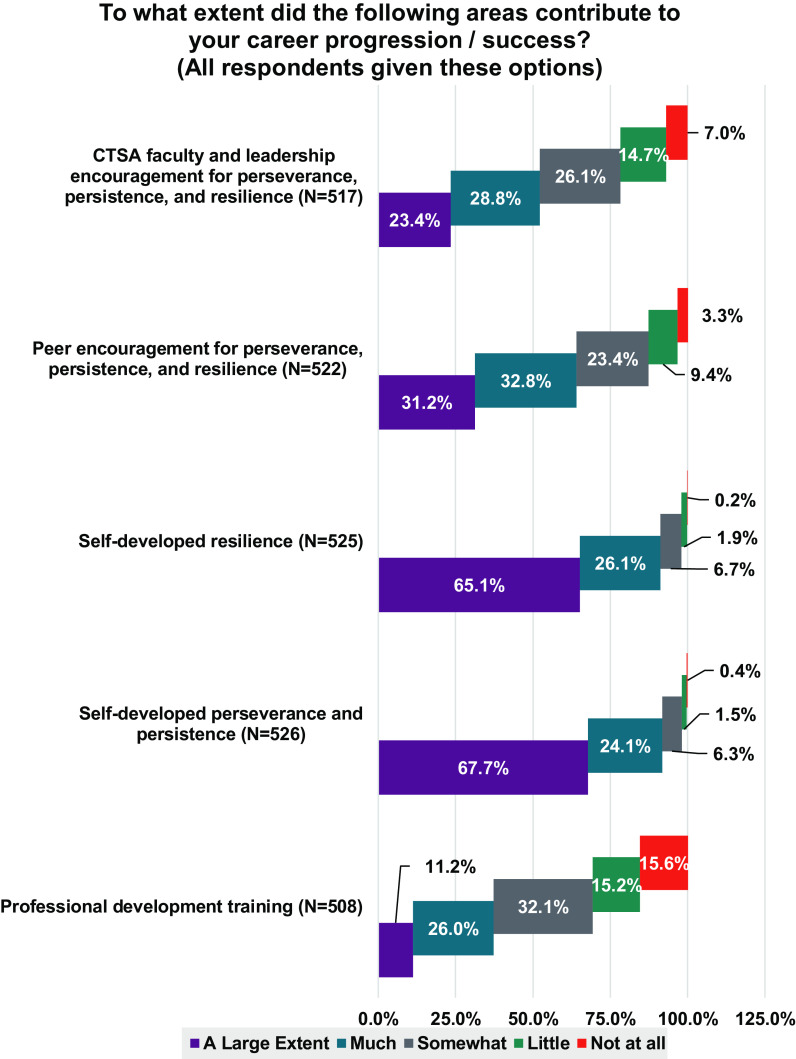
Factors that contributed to KL2 scholar success. The results are presented as the percent of respondents that perceived each area as contributing a large extent (purple), much (turquoise), somewhat (gray), little (green), or not at all (red) to their career progression and success. The number of responses for each area is indicated by N. Abbreviations: Clinical and Translational Science Award (CTSA).

During the KL2 award period, many structured opportunities were made available to Scholars and were utilized at different rates (Fig. 2), with certain ones perceived as having contributed to their career success (Fig. 3). Factors that had high utilization and were noted by Scholars as contributing to “a large extent” or “much” to career success were: protected time; mentoring; collaborations; grant writing training; pilot grants (beyond KL2); mock review of proposals; peer mentoring; and, research support staff and services. Self-developed resilience and perseverance were also noted by a majority of Scholars as contributing to a “large extent” or contributing “much” to their career success (Fig. 4). Likewise, more than half of the respondents considered encouragement by CTSA faculty, leaders, and peers to be important contributors to their success.

Of the alumni who incurred debt to fund their education, 59.0% indicated that debt did not influence their ultimate career choice, 25.9% felt that their debt “highly influenced” or “somewhat influenced” their career direction, while 15.1% were neutral about the impact of their debt on their career. Most KL2 alumni (57.2%) did not apply for the NIH loan repayment program; of the 42.8% who did apply, 33.0% received loan repayment support. Of those who utilized the loan repayment program, 84% felt that it contributed to a “large extent” or “much” to their career progression and success.

In open-ended responses, KL2 alumni specified challenges they faced, professional experiences they found especially beneficial, or other factors that influenced their career progression (Table 1). Barrier and facilitator themes comprised mentorship, leadership, administration; money, funding, salary, debt; time to complete research; work environment; networking, connections; work/life balance; workload; and KL2 program content. Approximately half (52.4%) of the comments were coded as negative or barriers, while a fourth were positive or as facilitators (23.6%) or neutral (24.0%). Analysis of the qualitative responses reinforced the value of protected time, mentorship, networking, and adequate research funding (see Table 1 and Supplementary Data). Concerns raised by the KL2 alumni included problems with mentorship and leadership, research funding challenges, and the inability to integrate work and life responsibilities.

**Table 1.** Career satisfaction responses

Are there any other comments or feedback about the challenges you have faced, or professional experiences that you found especially beneficial, or other factors that influenced your career progression? (N = 154) (Cont.)		
Theme	N^*^	Example comments
Mentorship, leadership, administration	65	*The one thing that has helped me a lot is having several mentors from various disciplines.*
		*mentorship is key*
		*A lot of expectations and goals are set for early career, young investigators – which seems set in place 2 decades ago (when getting NIH grant (R01) or Renewal of an NIH grant was possible with just one submission). These same rules are being applied by those so-called "experienced professors" who are part of the committees or even chairs assessing young investigator’s achievements within merely 3 years of appointments. This routine is posing A LOT of stress on young investigators (who might have young children, balancing their family and professional life). NIH and NCATS needs to pay a particular emphasis and send a MESSAGE to such institutions to reduce unrealistic expectations and stress on young investigators. This would reduce migration of recently graduated PhDs or Postdocs to the industries. (Please DO NOT propose stress-reducing, balancing the life-work blah blah sort of courses – these are waste of time out of our busy teaching, research and family schedules!).*
Money, funding, salary, debt	42	*By my primary mentor telling me that they have a large pile of unfunded grants, this helped me a great deal to realize the need for persistence and continuing to seek grant funding as well as conducting research which could lead to grant funding.*
		*I just want to mention that i did not apply to the NIH Loan repayment program because i had no student debt, perhaps that should be an option in future surveys*
		*Physicians need a chance to do research! Research is more and more competitive to get grants. We submit grants and they get held up or tore apart by non-clinicians that don't understand the impact or clinical side of research. Overly-competitive, overly-critical comments is all that is received. Mice immunologists get funded through out the country to do esoteric, non-translational experiments while many important translational scientists are denied funding and pressured into more and more clinic time. It’s a terrible and broke system.*
Time to complete research	17	*protected time from the KL2 was by far the single biggest contributor to my success*
		*Protected time*
		*the protected time for research is not necessarily protected well from administrative and teaching within the institution. I thought my mentor did a good job of emphasis on protection from clinical obligation but the rest was not well protected and easy to get overextended, lose focus on research.*
Work environment	13	*Being part of women’s peer mentoring group was very beneficial and gave me the confidence for ask for what I deserved. We discussed topics like negotiation, imposter syndrome, networking and childcare. It was nice to talk with other like-minded women in science/academia who were going through similar struggles.*
		*Translational science has a lot of unknowns but opportunities will present themselves and young researchers need to be courageous and Universities need to recognize and reward courage and alienate young researchers or risk having the good ones transfer and lose gratitude for all the University has done for them.*
		*The KL2 program needs to aware of gender inequality (even at [institution]); I have been in a meeting, and clearly been spoken down to because of my gender (not by a KL2 faculty, but an invited faculty at a KL2 meeting). I should of spoken up; but was somewhat shocked at others sat quietly while this man "made fun of me." This is no longer acceptable.*
Networking, connections	12	*The network that I built throughout my experience as an academic researcher helped springboard me for success in industry.*
		*Having centralized opportunities to interact with other scholars in a longitudinal manner would be helpful. Similarly, having knowledge of broader mentorship opportunities would be helpful.*
		*No opportunities for tenure or advancement at my current institution. Tenure and advancement is only set up for faculty instructors.*
Work/life balance	27	*I strongly encourage others to participate in the National Center for Faculty Development and Diversity’s Faculty Success Program (FSP). I really struggled immensely with the transition from fellow to faculty, but the FSP program introduced me to a new mindset of better work-life balance and increased research productivity. I have a much improved outlook of my career longevity now.*
		*[…]the quest for "work/life balance" seems it is misleading. One should strive at being the best at work and at family life, but not at what balance implies, a 50-50 proposition. Aiming for that is a stressful, elusive quest.*
		*I have a balanced life but only after years of imbalance that made me sick. My illness motivated me to seek balance.*
Workload	14	*overall satisfied; tough at times to juggle different roles, but feel much happier straddling multiple areas than going all-in in one area. would not do it differently if starting over now*
		*KL2 funds are not sufficient to help hire people to help with the projects, so the projects themselves need to be doable by the PI alone, somehow.*
		*I think my biggest career mistake is that I am spread across too many different realms. I have a heavy clinical workload, administrative roles, and my research roles are too varied.*
KL2 program content	28	*The opportunity was outstanding, and I am still able to do meaningful research because of this program (although I am primarily in private practice). I just couldn't afford to stay in full time research due to financial and family obligations.*
		*The KL2 Scholars program has been an invaluable experience that catapulted my career. But by same token, I think "what you get out of it is what you put into it" - success seems to be defined by whether or not candidates fully engage in the opportunities made available to them.*
		*Politics at academic institutions can outweigh documented achievements. The KL2 program should help its trainees be promoted and achieve tenure. Otherwise, the program is just a waste of Tax Payer money and is just for appearance that something is being done to help junior faculty.*
Other	36	*You have to enjoy and focus on the process, not the outcomes*
		*This survey is poorly designed. Q32 assumes I am a clinician but leaves me no option to respond as a non-clinician. Likewise, for 27-31, I incurred zero educational debt but there was no option to respond as such for those questions.*
		*This is an extremely challenging career choice. Most days I am fulfilled by my work and glad I am continuing on this path. However it is not clear to me that there is sufficient support at the U to maintain a pipeline of junior faculty who will become independent physician-scientists in the future.*

* N = The number of times comments addressed the corresponding theme; exceeds number of respondents.

A table of all responses can be found in Supplementary Materials.

Comments appear as submitted, identifying information is redacted in [brackets].

## Discussion

This survey of KL2 alumni provides valuable insight into the perceptions of KL2 Scholars and their outcomes and experiences during and after their KL2 award period. The data suggest that the large majority of Scholars continue to be engaged in clinical and translational science years after completing the program. Important themes that emerged were: (i) the KL2 program resulted in a high level of Scholar participation in research, irrespective of terminal degree; (ii) Scholars noted that the KL2 program was very beneficial for their career progression; and (iii) Scholars were satisfied with career choice and direction, national recognition, and impact of their work across the entire translational science spectrum. Career satisfaction was similar irrespective of gender or professional/doctoral degree type. Importantly, half of the KL2 alumni reported attaining leadership positions.

One limitation to the survey is that only 25% of alumni responded and those who completed the program many years prior had lower response rates. Thus, results may be biased with respect to the characteristics of the individuals who chose to respond. In that vein, 95% of the KL2 respondents in our survey reported still being in academics, whereas a 2017 survey of KL2 program directors indicated that 76% of the KL2 Scholars were performing translational science at a CTSA institution [1]. Likewise, the rates of extramural funding success may be due to a reporting bias, i.e. KL2 alumni with funding were more likely to complete the survey. Nevertheless, our sample size of 547 is robust by faculty survey standards and a response rate of 25% is in line with other survey methods.

Although Scholars participated in a robust set of academic and career development offerings during their KL2 training, the most cited factor contributing to career success was the protected time provided by the KL2 program. These findings support the importance of protecting a substantial proportion of time (50–75%) for research for early career professionals. Mentorship and collaborations also contributed substantially to success. The quantitative data were not collected in a manner that distinguished between KL2-program driven efforts to promote collaborations (e.g. focus on team science) and those that formed organically. A deeper analysis of the qualitative feedback could provide more insight. Other factors that Scholars perceived as contributing to their success were self-identified personal attributes of resilience and perseverance. Our findings complement the data from structured interviews reported by Robinson and colleagues [3], who found that some KL2 Scholars perceived resilience as a personality trait, whereas most thought it was a skill learned by modeling parents, collaborators, and mentors. In this study, feeling connected to peers also was perceived as promoting resilience and persistence. Similar to our findings, Robinson and colleagues reported varying views about work and life integration, with 62.5% of their respondents reporting difficulty in achieving work/life balance [3]. Two strategies to lessen the stress reported by KL2 Scholars were seeking balance over an extended period of time, rather than on a daily basis, and lowering expectations.

The KL2 respondents identified missed opportunities that can help improve career development programs. In open-ended responses, they noted the underutilization of potentially beneficial program elements such as the loan repayment program, budget management training, leadership training, formalized professional and grant writing training, research support services, access to core facilities, and peer mentorship/collaboration opportunities. Concerns articulated by Scholars about work/life integration indicate a need to continue to refine strategies to address this key issue. Additionally, a challenge for the medical community is how to address misgivings about the long-term financial sustainability of translational science as a career choice and the challenge of combining clinical care, teaching, and research. This challenge has become even more intense since the onset of the coronavirus 2019 (COVID-19) pandemic. NIH recently launched a Small Grants Program for KL2 recipients (See: https://grants.nih.gov/grants/guide/pa-files/PAR-21-121.html) that provides new opportunities for collecting additional pilot or feasibility data that can support grant applications critical for sustaining the careers of this cohort.

The KL2 Scholar survey responses highlight the importance of collaborations on career success. Additional work needs to be done to understand whether specific KL2-promoted activities meaningfully improve the perception of collaborations, the number of collaborations, and/or correlation of collaborations with indicators of career success. As examples, CTSA programs have historically emphasized team science and many hubs offer specific training in team science. More recently, KL2 programs have instituted efforts to stimulate cross-hub collaborations through a visiting scholars program [12]. The impact of these and other deliberate efforts to stimulate mentoring and collaborations should be assessed.

Our findings of the research funding success of the KL2 cohort are in keeping with studies of other career development programs. A recent analysis of the impact of NIH Individual Mentored Career Development Awards including K01, K08, and K23 awards, found that individuals who received K awards were more likely to obtain a first independent NIH award and have more awards than those who did not have a K award [13]. These findings indicate that mentored awards are important for launching an independent science career for early-career scientists. An analysis of the National Cancer Institute’s Career Development (K) award program in 2012 found that K award recipients were more likely to receive subsequent NIH grants, had greater numbers of publications, and were more likely to have a funded research career than individuals who applied for, but did not receive a K award [14]. Similar results have been observed for individuals supported by national foundation or society-career development awards [15]. Our findings are also in keeping with those previously reported from analyzing administrative datasets including eRA Commons and Information for Management Planning Analysis and Coordination II (IMPAC II) for the time frame 2006–2014. This prior study demonstrated that 46% of previous K awardees were successful in receiving grant support as PIs from federal agencies including NIH, Centers for Disease Control and Prevention, FDA, Agency for Healthcare Research and Quality, and Department of Veterans Affairs [16]. As translational science is team science, not surprisingly, 60.2% of KL2 Scholars served as co-investigators.

This survey of KL2 alumni was completed before the COVID-19 pandemic and the simultaneous heightened awareness and call to action regarding racial equity, diversity, and inclusion [17]. As a result, the impact of those events is not captured in the KL2 responses. A survey of current CTSA KL2 Scholars and TL1 trainees [18] shortly after COVID-19 was declared a pandemic indicated that they experienced a period of limited or restricted access to research facilities, clinics, and research participants. Those limitations adversely affected research productivity by some trainees, whereas, paradoxically, other trainees reported that the pandemic afforded them more time to think, write, and analyze data. That survey also found that trainees suffered from limited access to mentors and team members and faced added time pressures from the need to homeschool children. Many medical centers have had to enlist the clinical expertise of KL2 Scholars in treating the overwhelming number of patients with COVID-19, causing interruptions in protected KL2 time. Moreover, KL2 Scholars who are Black, Indigenous, and People of Color (BIPOC) likely experienced additional stress as racial inequities became more transparent. In our study, approximately 15% of the respondents self-identified as: Blacks or African Americans; Hispanics or Latinos; American Indians or Alaska Natives; Native Hawaiians, and other Pacific Islanders. In response to concerns raised by the biomedical community, NIH has issued COVID-19 flexibilities for NIH career development awardees [19]. Given the critical importance Scholars place on resilience and perseverance for career success, it will be important for future studies to compare these qualities to see whether the stresses of COVID-19 and racial inequities have long-lasting impacts on careers of KL2 Scholars.

Those limitations notwithstanding, this survey reports a high level of satisfaction with careers in clinical and translational science with high retention rates. It also demonstrated how the key attributes of KL2 programs are viewed by Scholars to have contributed markedly to their career productivity. Additionally, the open-ended responses from the survey will be available to the community for further analysis and should provide an important data asset for the community. Our survey results have implications for early-stage investigators funded by other types of career development programs offered by NIH and other funding agencies. Protected time, mentoring, and collaborations were critical facilitators for KL2 Scholar career success that likely apply to other early-stage investigators. Thus, addressing the factors KL2 Scholars cited as contributing to their career success, including protected time, mentoring, and collaborations are likely to have a widespread positive impact on the careers of the biomedical research workforce and thus for the biomedical science community in general.

## Supplementary material

For supplementary material accompanying this paper visit https://doi.org/10.1017/cts.2021.886. click here to view supplementary material

## References

[1] Sorkness CA , Scholl L , Fair AM , Umans JG. KL2 mentored career development programs at clinical and translational science award hubs: practices and outcomes. Journal of Clinical and Translational Science 2020; 4(1): 43–52. DOI 10.1017/cts.2019.424. 32257410 PMC7103475

[2] Sweeney C , Schwartz LS , Toto R , Merchant C , Fair AS , Gabrilove JL. Transition to independence: characteristics and outcomes of mentored career development (KL2) scholars at clinical and translational science award institutions. Academic Medicine 2017; 92(4): 556–562. DOI 10.1097/acm.0000000000001473. 28351069 PMC5373479

[3] Robinson GF , Schwartz LS , DiMeglio LA , Ahluwalia JS , Gabrilove JL. Understanding career success and its contributing factors for clinical and translational investigators. Academic Medicine 2016; 91(4): 570–582. DOI 10.1097/acm.0000000000000979. 26509600 PMC4811729

[4] Harris PA , Taylor R , Thielke R , Payne J , Gonzalez N , Conde JG. Research electronic data capture (REDCap)--a metadata-driven methodology and workflow process for providing translational research informatics support. Journal of Biomedical Informatics 2009; 42(2): 377–381. DOI 10.1016/j.jbi.2008.08.010. 18929686 PMC2700030

[5] Cottler LB , Green AI , Pincus HA , McIntosh S , Humensky JL , Brady K. Building capacity for collaborative research on opioid and other substance use disorders through the Clinical and Translational Science Award Program. Journal of Clinical and Translational Science 2020; 4(2): 81–89. DOI 10.1017/cts.2019.441. 32313696 PMC7159806

[6] McCormack WT , Bredella MA , Ingbar DH , et al. Immediate impact of the COVID-19 pandemic on CTSA TL1 and KL2 training and career development. Journal of Clinical and Translational Science 2020; 4(6): 556–561. DOI 10.1017/cts.2020.504. 33942017 PMC7605410

[7] McIntosh S , Wall AF , Johnson T , Kurtzman J , Ververs D , Ossip DJ. Tobacco control at community colleges: context and opportunities. Tobacco Prevention & Cessation 2016; 2: 76. DOI 10.18332/tpc/66949. 29218328 PMC5716631

[8] Block RC , Duron V , Creigh P , McIntosh S. International service and public health learning objectives for medical students. Health Education Journal 2013; 72(5): 530–536. DOI 10.1177/0017896912450874. 24489401 PMC3904446

[9] DeAngelis EJ , McIntosh S , Ahmed CD , Block RC. Familial hypercholesterolaemia patient-determined themes for community-engaged research. Health Education Journal 2017; 77(3): 293–302. DOI 10.1177/0017896917745567.

[10] Campbell JL , Quincy C , Osserman J , Pedersen OK. Coding in-depth semistructured interviews: problems of unitization and intercoder reliability and agreement. Sociological Methods & Research 2013; 42(3): 294–320. DOI 10.1177/0049124113500475.

[11] *Underrepresented Populations in the U.S. Biomedical, Clinical, Behavioral and Social Sciences Research Enterprise*. National Institute of Health, 2020. (https://diversity.nih.gov/about-us/population-underrepresented)

[12] Robb SL , Kelly TH , King VL , Blackard JT , McGuire PC. Visiting Scholars Program to enhance career development among early-career KL2 investigators in Clinical and Translational Science: implications from a quality improvement assessment. Journal of Clinical and Translational Science 2020; 5(1): e67. DOI 10.1017/cts.2020.564. 33948286 PMC8057478

[13] Nikaj S , Lund PK. The impact of individual mentored career development (K) awards on the research trajectories of early-career scientists. Academic Medicine 2019; 94(5): 708–714. DOI 10.1097/acm.0000000000002543. 30520806

[14] Mason JL , Lei M , Faupel-Badger JM , et al. Outcome evaluation of the National Cancer Institute career development awards program. Journal of Cancer Education 2013; 28(1): 9–17. DOI 10.1007/s13187-012-0444-y. 23292841 PMC3608862

[15] Safdar B , Paradise SA , McMillian M , Holmes JF. Influence of society for academic emergency medicine grant mechanisms on postaward academic productivity. Academic Emergency Medicine 2015; 22(2): 150–156. DOI 10.1111/acem.12571. 25641380

[16] Nagel JP. Historical assessment of the CTSA program KL2 scholar program (2006–2014). In: Translational Sciences Meeting. Washington DC: Google Scholar, 2018.

[17] Collins FS , Adams AB , Aklin C , et al. Affirming NIH’s commitment to addressing structural racism in the biomedical research enterprise. Cell 2021; 184(12): 3075–3079. DOI 10.1016/j.cell.2021.05.014. 34115967

[18] Health NIo. *Frequently Asked Questions (FAQs) COVID-19 Flexibilities*. (https://grants.nih.gov/faqs#/covid-19.htm?anchor=question55752)

[19] National Center for Advancing Translational Sciences. National Institute of Health. (https://ncats.nih.gov/)

