# Supplementary material for: KL2 scholars’ perceptions of factors contributing to sustained translational science career success
Source: J Clin Transl Sci. 2021 Dec 28;6(1):e34. doi: 10.1017/cts.2021.886 (PMC9003634; doi:10.1017/cts.2021.886)
Supplement: Supplementary file 1 [file S2059866121008864sup001.docx]

Supplementary Materials

|  | | |
| --- | --- | --- |
| **Demographics** | | |
| **Theme** | **%** | **Responses** |
| **Gender** | **39.3** | *Male* |
|  | **60.0** | *Female* |
|  | **0.80** | *No answer or self-described* |
| **Underrepresented minority** | **15.6** | *Yes* |
|  | **84.4** | *No* |
| **Disadvantaged Group** | **9.2** | *Yes* |
|  | **90.8** | *No* |
| **Disability** | **1.1** | *Yes* |
|  | **90.8** | *No* |
| **Terminal Degree** | **47.2** | *MD* |
|  | **36.1** | *PhD* |
|  | **12.8** | *MD-PhD* |
|  | **2.2** | *Other Clinical* |
|  | **1.7** | *Other Research* |
| **Academic Title (When starting KL2)** | **65.3** | *Assistant Professor* |
|  | **17.4** | *Instructor* |
|  | **8.6** | *Research Assistant Professor* |
|  | **8.6** | *Other* |
| \| **Did you pursue / obtain a degree or a certificate during your KL2 Funding period?** \| \| --- \| | **38.7** | *Yes* |
|  | **61.3** | *No* |
| **If yes, which type of degree** | **80.3** | *Masters* |
|  | **8.5** | *PhD* |
|  | **10.4** | *Certificate* |
|  | **0.9** | *Other* |

|  | | |
| --- | --- | --- |
| **During the KL2 award period, which opportunities do you wish had been available to you either through the KL2 program or through your institution?** **(N=233)**  *(Comments appear as submitted, identifying information is redacted in [brackets])* | | |
| **Theme** | **N*** | **Example Comments** |
| **Budget Management Training** | **8** | *Budget development and grant logistical management* |
|  |  | *Additional training in financial management/budgets (i.e. budgets for grants, managing personnel/salaries) as well as […]* |
|  |  | *Management of research teams, budgets.* |
|  |  | *[…] budgeting, financial skill trainng* |
| **Mentorship** | **34** | *mentorship, […]* |
|  |  | *Opportunities for mentoring, networking, […]* |
|  |  | *Having a mentoring team, rather than primary mentor; […]* |
| **Development of an Individual Development Plan (IDP)** | **3** | *Mock reviews Individual career development planning* |
|  |  | *Individual development planning with greater input from individuals outside of my mentoring team.* |
|  |  | *Not much but maybe more emphasis on an IDP that was integrated and vetted with my department division KL2 mentoring committee and tenure committee. Somehow integrating and aligning all these mentorship activities and goals would be best.* |
| **Leadership Training** | **26** | *Leadership training, project management* |
|  |  | *More PI leadership hands on skills.* |
|  |  | *Professional and leadership development* |
|  |  | *More leadership training* |
| **Innovation or Entrepreneurship training** | **1** | *Programs focused on public speaking and more exposure to [university name entrepreneurship program]. I also think that if there were regular meetings with KL2 peers & faculty for proposals would've been helpful. Most of the grant writing/mock reviews I received from my MPH classes prior to the KL2.* |
| **Didactic Training in Clinical and Translational Science** | **1** | *Didactic training in Clinical and Translational Science* |
| **Pilot Grants** | **6** | *Specific pilot grant funding for KL2 scholars* |
|  |  | *Pilot funding* |
|  |  | *Pilot grants* |
| **Professional Development Training** | **31** | *Formalized professional development tied into KL2* |
|  |  | *1. skills on how to negotiate and ask for resources […]* |
|  |  | *Career coaching, […]* |
|  |  | *[…]Professional development specific to lab management, how to build up a lab, etc.* |
| **Grant-Writing Training (e.g., Courses, K Club)** | **17** | *Grant review and grant writing coaching […]* |
|  |  | *I think more peer writing and grant-writing programs would have been helpful, and […]* |
| **Mock Review of Your Proposals** | **8** | *Required review of grant proposals.* |
|  |  | *Mock reviews […]* |
|  |  | *I do not remember availability of mock grant proposal reviews during my time in the program. That would have been immensely helpfu.* |
| **Grant Application Support** | **8** | *I would have liked more access to support staff to help with manuscript and grant writing. […]* |
|  |  | *Someone to help navigate […] and grant writing. Not a ghost writer, but essentially, an "advanced editor."* |
|  |  | *More grant writing support;* |
|  |  | *External grant review* |
| **Statistical Training** | **5** | *Formal training in bioinformatics/data science.* |
|  |  | *Access to informatics training* |
|  |  | *bioinformatics course* |
| **Opportunities for Funding** | **3** | *More research funds* |
|  |  | *Post K grants* |
|  |  | *Insurance of bridge funding if a federal k-level of higher award did not fund before it's end and […]* |
| **Work/Life Balance** | **6** | *[…] Self-care initiatives* |
|  |  | *Perhaps more guidance regarding career planning with a family* |
|  |  | *[…] and stress management* |
| **Research Support Staff and Services (e.g., BERD/stats design, access to cores and services)** | **21** | *Regulatory support/training (e.g., IND preparation)* |
|  |  | *stronger logistical/research admin support for my grant-funded work so I could spend more time on mid-range and longer term planning of next submissisons* |
|  |  | *[…]Reliable and timely Biostats service* |
| **Protected Time** | **13** | *Protected time to work strictly on submitting external grant proposals.* |
|  |  | *[…] Many trainees are not protected equally and the program, in my opinion, left the interpretation of "protected" time up to the chief or chair for that trainee. It puts trainees in a difficult position to try to demand this "protected time." Often, the chair says one thing and the chief says something else. Protected time should be mandated, enforced, and CLEARLY defined (ie, 75% means 6-8 weeks in some divisions, and 10-12 weeks in some divisions, etc--that time difference easily adds up and makes a difference).* |
|  |  | *[…] and guarantee of protected research time if this did not happen. […]* |
| **Editorial/Manuscript Assistance** | **7** | *Opportunity to better understand the peer review process at NIH* |
|  |  | *Writing groups for accountability* |
|  |  | *Editing services […]* |
| **Time Management Training** | **9** | *time management/[…] for pi's* |
|  |  | *More training in time management and […]* |
|  |  | *Peer-to-peer coaching of how to manage […] and time better. […]* |
| **Project Management** | **11** | *project management […]* |
|  |  | *instruction on managing a study team; […]* |
|  |  | *Management of research teams, […]* |
| **Peer Mentorship /Peer Collaboration/ Collaboration** | **27** | *more peers doing research using the types of methods i use (health services research / clinical research) […]* |
|  |  | *We had one year of focused coursework as a KL2 group, then we scattered. I missed the continued peer interaction. [...].* |
|  |  | *Peer-to-peer coaching of how to manage non-research activities and time better.[…]* |
| **Networking** | **8** | *better connection and integration to Other faculty* |
|  |  | *More opportunities to reach out to Other KL2 Awardees from Other CTSAs.* |
|  |  | *[…] interactions with Other CTSAs* |
|  |  | *More organized mentoring and networking opportunities.* |
| **Supportive Environment** | **13** | *Tenure Track More Departmental Commitment* |
|  |  | *It would have been helpful to be blinded to the clinical work, and how the hospital valued that more than research (RVUs). It was easier for me to do clinical work and teach than it is for me to learn to be a salesman on a research project.* |
|  |  | *ways to analyze whether a research career is really a match for the individual (for me it's not)* |
|  |  | *1. Better avenues for implementation science at the institution level. There is a tendency for some in leadership to separate research efforts from clinical operations. 2. National and international platforms for building a reputation as a systems scientist. Some view systems science as shallow or superficial. Systems science efforts achieve depth via a strong transdisciplinary focus. I'm going through my promotion process right now and this is a sticking point for some.* |
| **Longer Program** | **2** | *More time - anOther two years would have been great* |
|  |  | *More time, e.g., 4-year KL2 would have been excellent* |
| **Externships** | **3** | *leadership training, externships* |
|  |  | *I feel externships/external collaborations are the most beneficial to the scholars and should be more strongly encouraged and supported. It has been these opportunities to learn new methodologies that have been most helpful in writing competitive grant applications and bringing novel methodologies back to my institution.* |
|  |  | *External rotations to Other academic institutions and industry. We had one industry (one day) visit but that was not enough.* |
| **Loan Repayment Program** | **1** | *[…] I think there should be more information/encouragement given to trainees about Loan Repayment Programs considering the heavy weight of debt on trainees and how this can influence career planning/directions. […]* |
| **Positive Experience, No Suggestion** | **13** | *Excellent program all around!* |
|  |  | *I was very happy w the resources available!* |
|  |  | *N/A. very satisfied.* |
| **Program Content Suggestion** | **4** | *bioinformatics course* |
|  |  | *The Master's program came later. I wish I could've take courses. Leadership training* |
|  |  | *courses and hands-on experiences in giving effective presentations (oral, powerpoint, etc)* |
| **Other** | **7** | *Many of the activities offered currently in the list, not available originally.* |
|  |  | *specific aims projected onto a screen and critiques by peers and leaders -establishes institutional investigators providing tips for success and collaboration* |
|  |  | *see above* |
|  |  | *Could not attend all leadership/extra workshops due to schedule conflicts. Recording was made available towards the end of my K and hope that is continuing.* |
| **N =The number of times comments addressed the corresponding theme; exceeds number of respondents* | | |
